# LRG1 Alters Pericyte Phenotype and Compromises Vascular Maturation

**DOI:** 10.3390/cells14080593

**Published:** 2025-04-14

**Authors:** Alexandra E. Hoeh, Jui-Hsien Chang, Ronja S. Mueller, Mark Basche, Alessandro Fantin, Anastasios Sepetis, Giulia De Rossi, Athina Dritsoula, Robin R. Ali, Patric Turowski, Stephen E. Moss, John Greenwood

**Affiliations:** 1 Institute of Ophthalmology, University College London, London EC1V 9EL, UK; 2 Ocular Cell and Gene Therapy Group, Centre for Gene Therapy and Regenerative Medicine, King’s College London, London SE1 9RT, UK; 3 Department of Biosciences, University of Milan, 20133 Milan, Italy

**Keywords:** leucine-rich alpha-2-glycoprotein-1 (LRG1), pericyte, blood–retinal barrier, angiogenesis, blood vessel maturation

## Abstract

Upregulation of leucine-rich alpha-2-glycoprotein-1 (LRG1) contributes to aberrant neovascularization in many different diseases. In contrast, LRG1 is not involved in developmental angiogenesis. Here, we investigated the vasculopathic properties of LRG1 by examining its effect on developing retinal blood vessels. By injecting recombinant protein or an expression vector into the mouse retina during vascular development, we showed that exogenous LRG1 reduces pericyte coverage and NG2 expression. It leads to diminished collagen IV sheathing, fewer adhesion and gap junctions, and reduced vessel calibre and vascular density. Moreover, in mouse retinae containing exogenous LRG1, the developing blood–retinal barrier remains more permeable with significantly higher numbers of transcytotic vesicles present in microvascular endothelial cells. These results reveal that exogeneous LRG1 is sufficient to interfere with the maturation of developing retinal vessels and drive vessel development towards a dysfunctional phenotype. These observations deliver further evidence that LRG1 is an angiopathic factor and highlight the therapeutic potential of blocking LRG1 in diseases characterized by pathogenic angiogenesis or vascular remodelling.

## 1. Introduction

Physiological angiogenesis during development, wound healing and reproduction, leads to the formation of an organized hierarchical network of vessels. During this process vessels undergo stabilization and maturation through mechanisms that include the recruitment and investment of pericytes, formation of the basement membrane and development of barrier properties [1,2]. Functional maturation of each vascular bed is orchestrated by intrinsic and local extrinsic cues that determine the specific vascular phenotype necessary to meet the needs of the local tissue [1]. In the central nervous system pericyte recruitment is recognized as a crucial step in vessel maturation as it is required for barrier development [2], including the downregulation of transcytosis and the concomitant formation of a functional blood–retinal and blood–brain barrier [3,4]. Furthermore, close endothelial cell—pericyte contact is a prerequisite for the production and deposition of extracellular matrix proteins, which form a supportive basement membrane [5].

In contrast to physiological angiogenesis, neovascularization that occurs in disease is generally abnormal with neovessels exhibiting poor pericyte coverage, an immature phenotype and an irregular and chaotic growth pattern [6,7]. These structural abnormalities result in compromised perfusion, increased permeability, an accumulation of interstitial protein and fluid, and a predisposition to hemorrhage [7]. The formation of such dysfunctional vessels frequently contributes to disease progression with the outcome dependent on the disease and the organ affected [7,8,9]. Why angiogenesis under disease conditions fails to replicate physiological angiogenesis is not fully understood but may be partly explained by failure of the stabilization and maturation process initiated and maintained through mechanisms such as endothelial cell-pericyte crosstalk. While many of the signalling interactions are well established [10], how they become corrupted in diseases remains to be fully elucidated.

In the search for angiopathic factors that contribute to neovascular dysfunction, the secreted glycoprotein leucine-rich alpha-2-glycoprotein-1 (LRG1) has been identified as a possible candidate which contributes to the formation of dysfunctional neovessels in retinal disease [11]. Further studies in animal models of disease confirmed its vasculopathic role [12] where it was reported to promote aberrant angiogenesis in corneal neovascularization [13], CNV [14,15], diabetic nephropathy [16,17,18], lung emphysema [19], bronchopulmonary dysplasia [20], cerebral ischaemia [21], and tumour vascularization [22,23,24]. In all models reported, LRG1 gene knockout led to the attenuation of disease progression [12], demonstrating its potential utility as a therapeutic target [8,15,25,26]. Importantly, high levels of LRG1 are seen in human diseases where pathogenic dysfunctional vessel growth, or abnormal vessel remodelling, occurs. Accordingly, LRG1 has been shown to be induced in patients with age-related macular degeneration [27], diabetic retinopathy [28], diabetic kidney disease [18], cancer [22,29,30,31], and cerebrovascular disease [32,33]. Many of these studies demonstrate that elevated LRG1 expression correlates with dysfunctional vasculature and worse disease outcomes.

To test the potential of targeting LRG1 as a therapy, several studies have shown that treatment with an LRG1-blocking antibody results in disease attenuation. In mouse models of retinal vascular disease and cancer, LRG1 inhibition reduces neovascularisation and vascular dysfunction [11,24], possibly by removing its vascular destabilizing effects. Indeed, LRG1 has been found to prevent vessel maturation through impairing the close spatial interaction between pericytes and endothelial cells [24,34], an arrangement that is crucial for vessel homeostasis [35].

In contrast to pathogenic vessel growth, the expression of *Lrg1* in the developing retina is believed to be negligible. In accordance with this, global *Lrg1* knockout does not result in a permanent vascular phenotype, demonstrating that only when *Lrg1* is induced in disease does it impact vessel structure and function [11]. Physiological vascular growth is mediated by several growth factors of which VEGF predominates, and crosstalk between the endothelial cells and pericytes coordinates their interactions, resulting in vessel stabilization and maturation [36]. The mouse retina vasculature develops postnatally and therefore has been used extensively to interrogate both physiological and pathological vessel growth [36]. In this study, we have introduced exogenous LRG1 to investigate its effect on physiological retinal angiogenesis. This approach aimed to deliver direct evidence that the presence of LRG1, in the absence of other confounding factors, disturbs normal vessel growth. We demonstrate that the exposure of developing vessels to LRG1 impaired pericyte coverage, disturbed the normal expression pattern of several pericyte markers, and compromised vessel maturation and function, indicating that LRG1 promotes vascular dysfunction and further supporting its potential as a therapeutic target in diseases characterized by abnormal vessel remodelling and growth.

## 2. Methods and Materials

### 2.1. Animals

The study included male and female mouse pups. Biological sex was not considered as a biological variable. C57BL/6 breeders were purchased from Charles River Laboratories. The NG2DsRedBAC transgenic mice which express DsRed under the control of the promoter for NG2 [37] were a generous gift from David Attwell (Jodrell Professor of Physiology, UCL). The *Lrg1*^−/−^ mice were generated on a C75BL/6N background by the knockout mouse project (KOMP) repository of the University of California, Davis. Mouse litters with their lactating mother were housed in transparent cages under environmentally controlled standard conditions and a 12 h light–dark cycle. Animals had free access to rodent chow and water.

### 2.2. LRG1 Protein

Recombinant human LRG1 protein was produced in-house as described earlier [11]. Pups were injected intravitreally on postnatal day 2 (P2) under short cryoanaesthesia with 0.4 µL of 5 mg/mL LRG1. Controls received either 5 mg/mL LRG1 protein which was denatured by boiling at 95 °C for 5 min or PBS. Eyes were harvested 3 days after LRG1 injection on postnatal day 5 (P5).

### 2.3. LRG1 Overexpression Vector

The AAV (adeno-associated-vector)-based overexpression vector for human *LRG1* (AAV7m8-huLRG1-IRES-tdTomato) was based on a pd10 vector backbone containing the AAV2 ITRs (inverted terminal repeats) and packaged within a 7m8 capsid [38]. Expression of the human his-tagged *LRG1* transgene was driven from a CMV (cytomegalovirus) promoter. To allow simultaneous expression of the reporter tdTomato from the same mRNA transcript, it was constructed as a bicistronic vector with an internal ribosomal entry site (IRES) (Supplementary Figure S1). Recombinant AAV (rAAV) vectors were produced via triple transient transfection of HEK293T cells, purified by affinity with an AVB Sepharose column (GE Healthcare, Piscataway, NJ, USA) and concentrated using Vivaspin 4 (10 kDa) concentrators (Vivaproducts, Littleton, MA, USA) as described [39]. Vector genome (VG)-based titers were determined by quantitative real-time PCR (qPCR), using an inverted terminal repeats (ITR) binding assay [40]. The null vector construct, which was used as the control, was also based on a pd10 backbone plasmid packaged within a 7m8 capsid containing the sequence for a synthetic construct retinitis pigmentosa GTPase regulator gene (NIH GenBank: KY293401.1) between the ITRs. It does not have a promoter and the first 16bp including the start codon were deleted. The pups were injected intravitreally on P2 under short cryoanaesthesia with either 0.4 µL *LRG1* overexpression vector (titre of 2–5^13^ vg/mL) or the AAV7m8 null vector. The eyes were harvested on P16, a time point chosen to allow sufficient *LRG1* expression and exposure of the tissues to LRG1.

### 2.4. Oxygen-Induced Retinopathy (OIR)

The offspring of heterozygous breeding pairs (*Lrg1*^+/−^) were used for OIR to be able to compare wild type and homozygous knockout (*Lrg1*^−/−^) littermates. Nursing mothers and their litters were exposed to 75% oxygen for 5 days from P7 to P12. The eyes were harvested on P16 and tissue was taken for genotyping. Only retinas from wildtype and knockout (*Lrg1*^−/−^) pups were used for immunohistochemical staining.

### 2.5. Vascular Leakage

C57Bl/6 pups received an intravitreal injection of LRG1 protein into one eye, and denatured LRG1 into the fellow eye on postnatal day 2. On P5, 30 µL of 1 mg/mL 10 kDa Dextran, Alexa Fluor™ 488 (D22910, Thermofisher Scientific, Waltham, MA, USA) or 1 mg/mL Albumin from Bovine Serum (BSA), Alexa Fluor™ 488 conjugate (A13100, Thermofisher Scientific) was injected into the left ventricle under terminal anesthesia, and the mice were culled after 10 min of continuous heartbeat. Enucleated eyes were immediately fixed in 4% PFA, dissected, fixed for another hour in PFA, and stained for podocalyxin to visualize the vasculature. Leakage was quantified in thresholded images of the retinal flatmounts by measuring the total pixel area of tracer extravasation per eye which was displayed as the absolute tracer-positive pixel number.

### 2.6. Immunohistochemistry

Retinal flatmounts were fixed for at least 20 min in −20 °C methanol or for at least 1 h in 4% PFA, and were incubated overnight with the following antibodies: rat monoclonal anti-CD31 (553370, BD Pharmingen, San Diego, CA, USA), rabbit polyclonal anti-CD31 (ab28364, Abcam, Cambridge, UK), rat monoclonal anti-CD31 (DIA-310-DIA, Stratech Scientific, Ely, UK), goat polyclonal anti-collagen IV (AB769, Millipore, Temecula, CA, USA), rabbit polyclonal anti-desmin (ab15200, Abcam), rabbit polyclonal anti-NG2 (AB5320, Millipore), rabbit polyclonal anti-connexin43 (C6219, Sigma-Aldrich, Saint Louis, MO, USA), rat monoclonal anti-VE-cadherin (550548, BD Pharmingen), rat monoclonal anti-PLVAP (550563, BD Pharmingen), rabbit polyclonal anti-MFSD2A (57755, Cell Signalling Technology, Leiden, The Netherlands), rat monoclonal anti-podocalyxin (192703, R&D systems, Minneapolis, MN, USA), goat polyclonal anti-podocalyxin (AF1556, R&D systems), mouse monoclonal anti-claudin-5 Alexa Fluor^®^ 488 conjugate (352588, Thermofisher Scientific, Waltham, MA, USA), mouse monoclonal anti-occludin Alexa Fluor^®^ 594 conjugate (331594, Thermofisher Scientific) and goat polyclonal anti-PDGFRβ (AF1042, R&D systems). Retinas were then incubated with the appropriate secondary antibodies Alexa Fluor^®^ 488, Alexa Fluor^®^ 594 or Alexa Fluor^®^ 647 (Invitrogen, Thermofisher Scientific) for 2 h, mounted in S3023h Dako fluorescent mounting medium and examined by confocal microscopy (LSM 710 confocal laser scanning microscope, Carl Zeiss, Jena, Germany).

### 2.7. Single-Cell RNA Sequencing Analysis

The mouse P6 retina dataset (GSE175895) was downloaded from GEO NCBI “https://www.ncbi.nlm.nih.gov/geo/ (accessed on 21 September 2021)” [41]. R Studio v.1.3.1056 and Seurat version 3.2.3 [42] were used to explore scRNAseq data. Cells containing less than 500 feature counts and genes expressed in less than 10 cells were removed. Downstream analysis included data normalization (“LogNormalize” method and scale factor of 10,000) and variable gene detection (“vst” selection method, returning 2000 features per dataset). The principal components analysis (PCA) was performed on variable genes, and the optimal number of principal components (PCs) was chosen using the elbow plot. The selected PCs were used for Louvain graph-based clustering. Uniform manifold approximation and projection (UMAP) was chosen as a non-linear dimensionality reduction method, and cluster cell identity was assigned by manual annotation based on known marker genes. Each relevant gene was then examined using the FeaturePlot and VlnPlot functions.

### 2.8. Transmission Electron Microscopy

Pups received intraocular injections with 7m8-LRG1 or 7m8-null on P2. At P10, they were injected intraperitoneally with 100 μL of 75 mg/mL horseradish peroxidase (HRP) and culled 4 h later. Eyes were immediately fixed in 4% PFA. After performing a DAB reaction (30 min incubation in 0.075% DAB/0.02% hydrogen peroxide in 0.1 M Tris, pH 7.6), the eyes were further fixed in Karnovsky solution, embedded in Epon resin, cut into 70 nm ultrasections with the Leica Ultracut UCT microtome, mounted on 150hex copper TEM grids and stained with Reynold’s lead citrate. To ascertain analysis of leading edge microvessels, eyes were sectioned from the anterior end and the first sections with clearly discernible microvessel lumens used for further analysis. Images of retinal vessels with a diameter <10 μm were taken with the JEM1400plus transmission electron microscope (JEOL, Welwyn Garden City, UK) equipped with an Orius CCD camera (Gatan Inc., Pleasanton, CA, USA) and analyzed using ImageJ (version 1.53a). HRP-filled vesicles in endothelial cells which were <100 nm in diameter and associated with the plasma membrane were quantified and normalized to the length of the endothelial plasma membrane in μm as described previously [43].

### 2.9. Analysis and Statistics

Before immunohistochemical analysis, images were masked by randomly assigning a new file name with either the free software ‘Bulk rename utility’ (version 3.3.2.0) or the help of an uninvolved colleague to conceal the treatment group. ImageJ (FIJI open source imaging processing software, version 1.53a) and Adobe Photoshop cs version 8.0.1 were used for image analysis. To measure pixel intensity, we created an individual mask per image to pick up whole cells independent of their brightness and then measured mean pixel intensity within this mask. Pixel intensity was not normalized to any other cell marker to avoid bias caused by concomitant LRG1 induced changes to these other markers. In the graphs, mean pixel intensity values are displayed normalized to the control. The semiautomated analysis of total tubule area and tubule junctions of the vasculature was performed with AngioSys 2.0 Image Analysis Software (Cellworks, Buckingham, UK). All analysis was based on *n* ≥ 3 experimental datasets with each experiment consisting of the eyes of one litter. Each data point in the graphs represents one eye unless indicated otherwise. Statistical analysis was performed with Prism version 6.01 (GraphPad, San Diego, CA, USA). After testing for normal distribution with the D’Agostini-Pearson omnibus normality test, the two-tailed Student’s *t*-test was used for normally distributed data. If the F test showed significantly different variances of the two groups, the *t*-test with Welch’s correction was used. For non-Gaussian data distribution, the Wilcoxon signed rank test or Mann–Whitney U test were used. Error bars represent standard errors.

## 3. Results

### 3.1. LRG1 Reduces Pericyte Coverage and Pericytic NG2 Expression in the Retina

**Figure 1:**
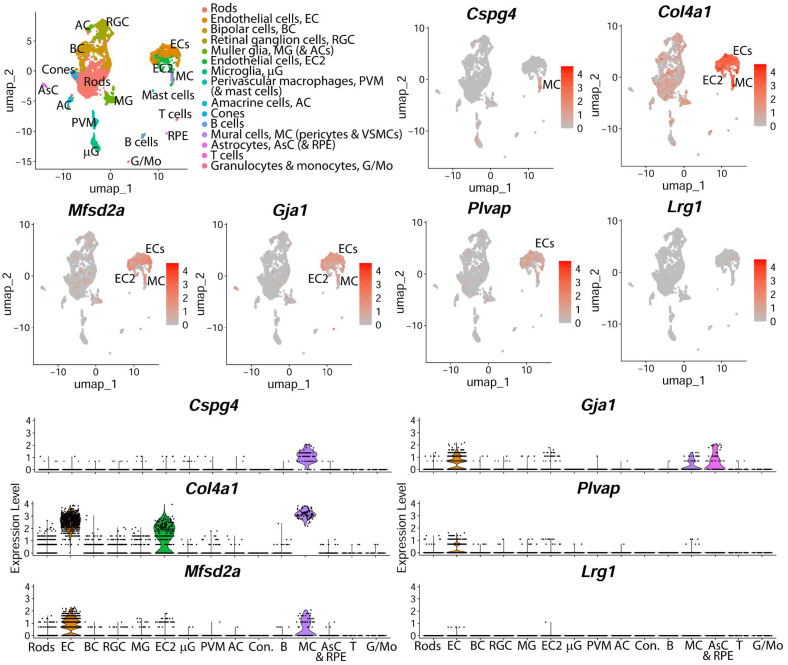
*Lrg1* is expressed at very low levels postnatally in the retina. Single-cell RNA sequencing data from mouse retina on postnatal day (P)6. A colour-coded UMAP of cell type identity is shown alongside UMAP illustrating the expression levels of the indicated genes per cell in each cluster. Each UMAP plot names the cluster(s) expressing the indicated gene. The corresponding violin plots illustrate the expression levels of each cell cluster. Each data point represents the value for one cell.

We first established that *Lrg1* expression in the retina during vascular development was negligible. Single-cell RNA sequencing (scRNAseq) analysis revealed minimal expression in retinal endothelial cells on postnatal day (P)6 and no *Lrg1* expression in any other retinal cells. In contrast, other genes (*Cspg4*, *Gja1*, *Col4a1*, *Plvap* and *Mfsd2a*) were expressed at significant levels in endothelial cells or pericytes (Figure 1). *Lrg1* gene expression remained low from P5 to P16 compared to expression in oxygen-induced retinopathy where pathogenic vessel growth takes place (Supplementary Figure S1).

**Figure 2:**
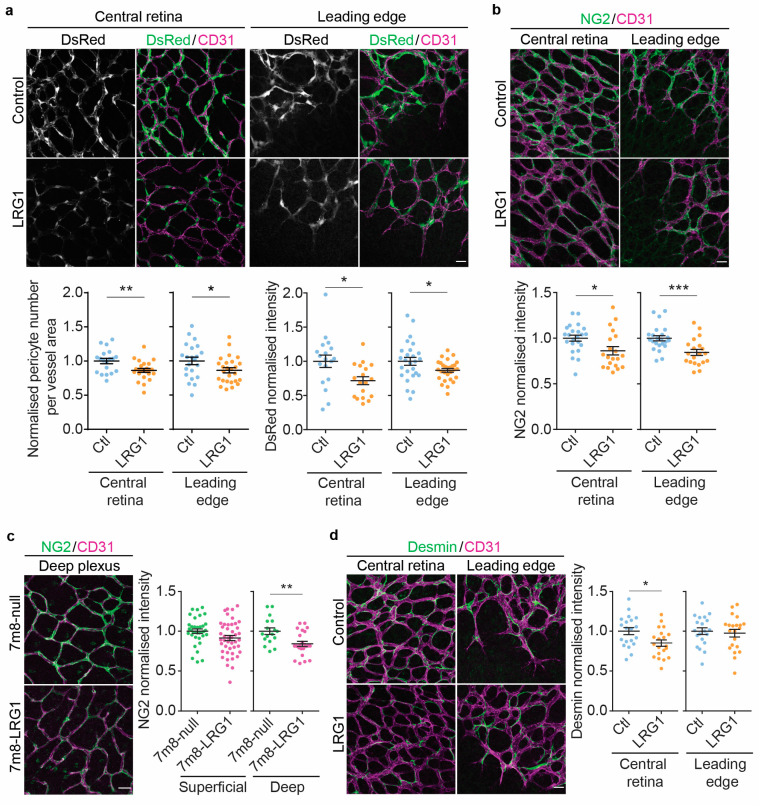
LRG1 alters the expression of pericyte markers and reduces pericyte coverage. (**a**) Representative micrographs of transgenic NG2 reporter mice expressing DsRed under the control of the NG2 promoter at P5. Pericytes were counted and normalized to the vessel area of each image, with lower pericyte density observed in LRG1-treated vessels; *n* = 19–26 eyes per group. Reduced expression of DsRed in pericytes at P5 after intravitreal injection of LRG1 protein; *n* = 18–28 eyes per group. (**b**) Representative micrographs demonstrating reduced expression of NG2 in microvascular pericytes of the central retina and at the leading edge of the growing vasculature in LRG1 protein-treated and control retinas. Quantitation shows significant differences between control and LRG1-treated retinas at P5; *n* = 20–23 eyes per group. (**c**) Representative images of the deep vascular plexus at P16 demonstrate weaker staining for NG2 in *LRG1* overexpressing retinas (7m8-LRG1) compared to null vector controls (7m8-null). Quantitation shows significantly reduced NG2 expression in the deep vessel plexus after injection of the *LRG1* overexpression vector and non-significant reduction in NG2 expression in the superficial plexus; *n* = 35–44 eyes per group (superficial plexus) and *n* = 17–23 eyes per group (deep plexus). (**d**) Images and dot plots showing reduced expression of desmin in capillaries in the retinal centre, but not the leading edge, at P5 following LRG1 protein treatment; *n* = 20–21 eyes per group. Unpaired *t*-test. Mean ± SEM of *n* ≥ 3 independent experiments. * *p* ≤ 0.05, ** *p* ≤ 0.01, *** *p* ≤ 0.001. Scale bars 25 μm.

Having demonstrated that *Lrg1* is expressed at very low levels postnatally in the retina, we next investigated if exogenous LRG1 introduced into the postnatal retina is capable of disturbing vessel formation. A single dose of recombinant human LRG1 protein was injected intravitreally on postnatal day (P)2. Three days later, at P5, the effect on vessel formation was investigated. To quantify changes in the number of pericytes associated with the retinal vasculature, we used transgenic mice that express DsRed fluorescent protein under the control of the NG2 promoter [37]. Following administration of LRG1 there was a significant reduction in the number of pericytes covering retinal vessels. In addition, we found a concomitant reduction in DsRed fluorescence in the LRG1 protein-treated retinas in this reporter model which was measured as pixel intensity within the DsRed positive area (Figure 2a). To confirm this effect, we then injected LRG1 protein into the eyes of C57BL/6 wild type pups and analyzed the expression of the pericyte marker NG2 (see *Cspg4*, Figure 1) in P5 retinal flatmounts. We observed a significant reduction in the expression of NG2 at both the leading edge of vascular expansion as well as in established vessels of the central retina (Figure 2b). To increase and extend the exposure of the retina to LRG1, we then injected an AAV7m8-hLRG1 overexpression vector into the eyes of P2 pups to transduce all nuclear layers of the retina and induce abnormally raised levels of extracellular LRG1 (Supplementary Figure S2). As observed following a single injection of LRG1 protein into the retina, AAV-mediated *LRG1* overexpression also resulted in reduced NG2 expression in pericytes on P16. The effect, however, was stronger in the deep vessel plexus than in the superficial plexus, where its reduction did not reach significance (Figure 2c).

Having established the effect of LRG1 on NG2 expression in the developing vasculature, we sought to determine the relevance of this finding in neovascular ocular pathologies where LRG1 expression is upregulated. To examine if the loss of *Lrg1* would increase NG2 expression in this setting, we compared hypoxia-induced neovascularization [44] in retinas from P16 wild type (WT) and *Lrg1*^−/−^ knockout oxygen-induced retinopathy (OIR) littermates. As observed previously in [11], *Lrg1* gene transcript was increased in the retina of WT OIR mice (Supplementary Figure S1) and *Lrg1* deletion resulted in a reduction in neovascular tufts of 15.4% (Mann–Whitney test, *p* ≤ 0.05, SEM = 5.4, 41–47 retinas per group). Expression of the pericyte marker NG2 revealed significantly stronger staining on the neovascular tufts and on the surrounding superficial capillaries of the *Lrg1*^−/−^ retinas (Supplementary Figure S3), confirming the effect of LRG1 on pericytic NG2. Our finding that LRG1 impacts NG2 expression and pericyte number suggests that, amongst other possibilities, LRG1 may disrupt the vasculature by affecting the intimate association between pericytes and endothelial cells.

Following this, we investigated if the introduction of exogenous LRG1 into the developing vasculature alters the expression of further pericyte markers. Consistent with the effects of LRG1 on NG2, expression of the intermediate filament protein desmin was also reduced in the central parts of the retina (Figure 2d). However, expression of platelet-derived growth factor receptor β (PDGFRβ), which is expressed by pericytes during vascular development, was not altered (Supplementary Figure S4).

### 3.2. LRG1 Reduces Vessel Collagen IV Coverage

**Figure 3:**
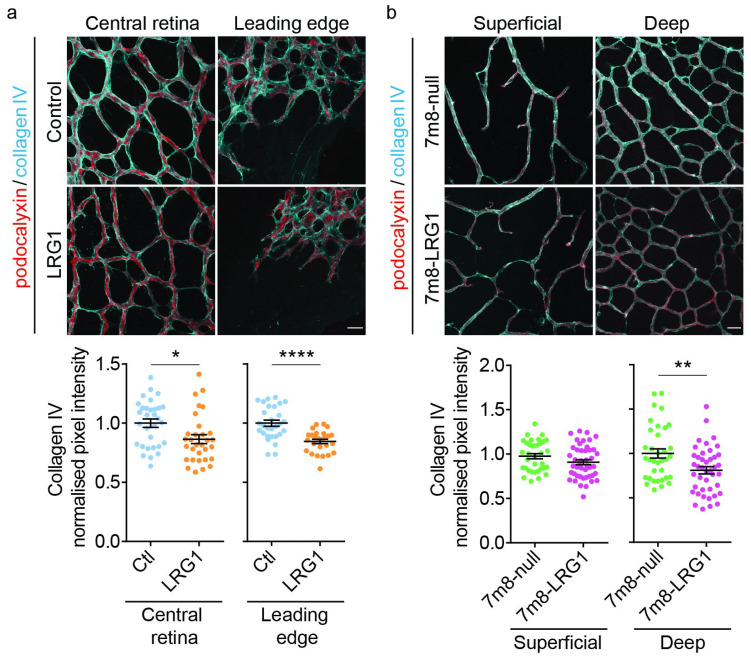
LRG1 impairs collagen IV ensheathing of retinal microvessels. (**a**) Illustrative micrographs of retinal vascular collagen IV expression at P5 following intravitreal injection with LRG1 protein or denatured LRG1 protein (control). Quantitation demonstrates reduced collagen IV expression in LRG1-injected eyes; *n* = 26–31 eyes per group. (**b**) Images of retinal vascular collagen IV expression at P16 following delivery of *LRG1* overexpression (7m8-LRG1) or null (7m8-null) vectors. Collagen IV expression was reduced in the deep vessel plexus in the *LRG1* overexpression vector group, but no difference was found in the superficial plexus; *n* = 34–45 eyes per group. Unpaired *t*-test or Mann–Whitney test was used depending on normality. Mean ± SEM of *n* ≥ 3 independent experiments. * *p* ≤ 0.05, ** *p* ≤ 0.01, **** *p* ≤ 0.0001. Scale bars 25 μm.

As pericytes are partly responsible for the deposition of the basement membrane, we investigated whether LRG1-induced pericyte reduction had any effect on the perivascular basement membrane. Following intraocular injection of LRG1 protein (Figure 3a) or AAV-mediated *LRG1* overexpression (Figure 3b) we analyzed the distribution of collagen IV, a basement membrane protein with significant expression levels in both endothelial cells and pericytes during retinal vascular development (see *Col4a1*, Figure 1). Introduction of LRG1 resulted in a significant reduction in collagen IV ensheathing of the developing microvessels. This was observed in vessels of the central retina and at the leading edge at P5 following recombinant protein delivery, and in the deep plexus at P16 following *LRG1* gene delivery. These data demonstrate that LRG1 decreases the amount of a crucial basement membrane component that is required for normal vascular function.

### 3.3. LRG1 Increases Vascular Leakage Without Altering the Expression of Tight Junction Proteins

**Figure 4:**
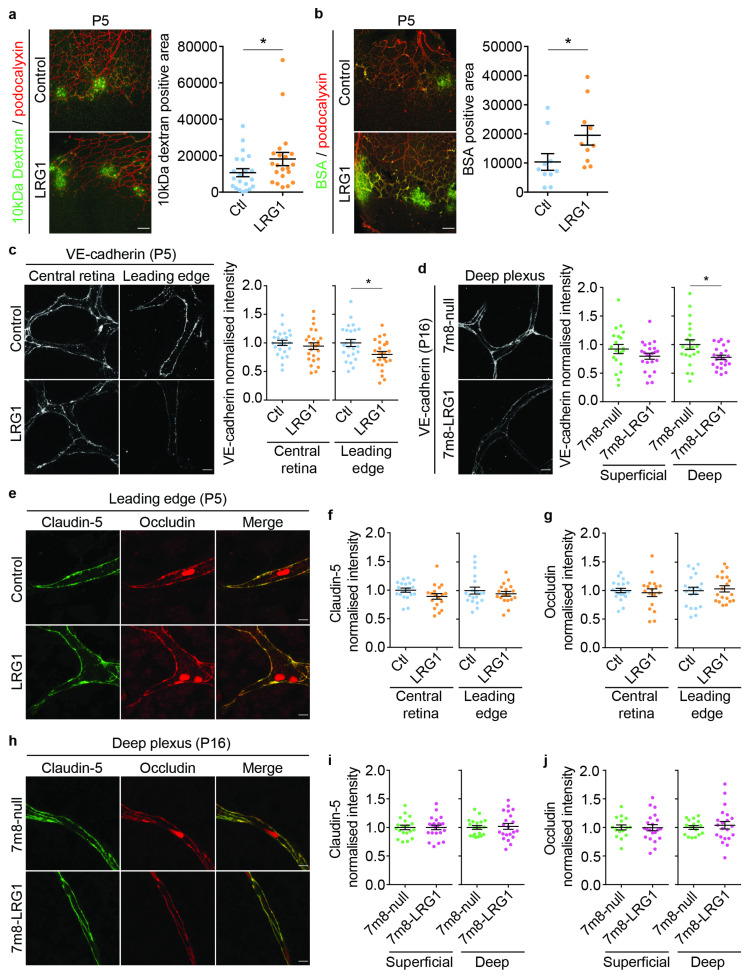
LRG1 increases vascular permeability without changing tight junction expression. (**a**) Images of Dextran-488 (10 kDa) and (**b**) bovine serum albumin (BSA-488) vascular leakage and associated quantification showing a significant increase in permeability at the leading edge of P5 eyes treated with LRG1; *n* = 21 eyes per group (Dextran-488) and *n* = 10 eyes per group (BSA-488). Scale bar 100 μm. Wilcoxon test. Mean ± SEM, * *p* ≤ 0.05. (**c**) Representative micrographs and quantified pixel intensity of junctional VE-cadherin showing reduced expression at the leading edge, but not in the central retina, of LRG1-treated eyes at P5; *n* = 24 eyes per group. Unpaired *t*-test. Mean ± SEM of *n* ≥ 3 independent experiments. * *p* ≤ 0.05. Scale bars 5 μm. (**d**) Significant reduction in VE-cadherin expression in the deep vessel plexus in *LRG1* overexpressing retinas (7m8-LRG1) at P16. No significant change was observed in the superficial plexus. *n* = 20–23 eyes per group. Unpaired *t*-test. Mean ± SEM of *n* ≥ 3 independent experiments. * *p* ≤ 0.05. Scale bars 5 μm. (**e**) Representative images of vascular claudin-5 and occludin expression at P5 following administration of LRG1 protein (scale bars 5 μm). Quantification revealed no change in expression of claudin-5 ((**f**); *n* = 18–19 eyes per group) or occludin ((**g**); *n* = 18–20 eyes per group). Bright luminal autofluorescent signal from erythrocytes was excluded from the analysis. Unpaired *t*-test. Mean ± SEM of *n* ≥ 3 independent experiments. (**h**) Representative micrographs of claudin-5 and occludin staining in the deep vessel plexus at P16 (scale bars 5 μm) following *LRG1* gene or empty vector delivery. Quantification revealed no change in expression of claudin-5 ((**i**); *n* = 19–22 eyes per group) or occludin ((**j**); 16–21 eyes per group) expression in the superficial or deep retinal vascular plexus. Unpaired *t*-test. Mean ± SEM of *n* ≥ 3 independent experiments.

As LRG1 affects pericyte association with endothelial cells, we then set out to determine whether it impacts retinal vascular permeability. Different molecular weight tracers, 10 kDa dextran and 67 kDa bovine serum albumin (BSA), were introduced into the circulation and retinal leakage accumulating within 10 min was determined by microscopic flatmount analysis. This revealed increased leakage of both tracers at the leading edge of vascular growth in LRG1-treated retinas when compared to controls (Figure 4a,b). To assess whether this might be due to an increase in paracellular or transcellular permeability or a combination of both, we next investigated if LRG1 affects the endothelial expression of junctional proteins. Vascular endothelial cadherin (VE-cadherin), which forms part of the endothelial adherens junction complex, is necessary for cell–cell adhesion and lumen formation, as well as the development of the blood–retinal barrier (BRB) and formation of tight junctions [45,46]. When compared to controls, treatment with LRG1 protein reduced the expression of VE-cadherin at the leading edge but not the central retinal vessels, and *LRG1* overexpression resulted in an attenuated signal in the deeper but not the superficial vessel plexus (Figure 4c,d), with the differences between the two approaches likely reflecting their impact on different stages of vascular development. We also observed that the expression of CD31, which is involved in cell–cell adhesion, leukocyte recruitment, the formation of vessel tubes and barrier function of the vasculature [47] was significantly reduced after LRG1 protein treatment at P5 (Supplementary Figure S5a). In *LRG1* overexpressing retinas, however, this was only observed in the superficial plexus, and not in the deep vessel plexus (Supplementary Figure S5b). As tight junctions are crucial in the regulation of paracellular transport, we then examined expression of the tight junction proteins claudin-5 and occludin. However, treatment with LRG1 protein or retinal *LRG1* overexpression did not affect the expression of either of these junctional proteins (Figure 4e–j).

### 3.4. LRG1 Alters the Expression of Genes Associated with Transcellular Transport and Increases Endothelial Vesicular Trafficking

**Figure 5:**
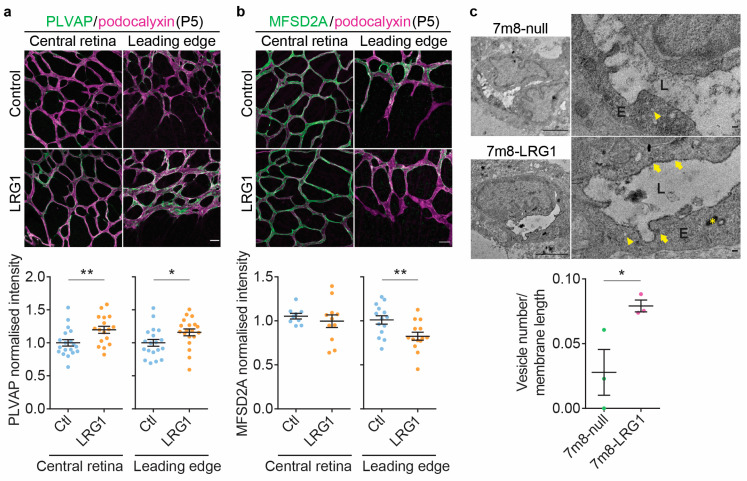
LRG1 increases transcellular transport at the developing BRB. Representative images and quantification of PLVAP (**a**) and MFSD2A (**b**) expression in retinal vessels at P5 following intravitreal injection of LRG1 protein. Quantification shows that LRG1 administration results in higher levels of PLVAP centrally and at the leading edge, and lower levels of MFSD2A expression at the leading edge compared to controls (scale bar, 25 μm); *n* = 17–19 eyes per group (PLVAP) and *n* = 9–14 eyes per group (MFSD2A). Unpaired *t*-test, mean ± SEM, * *p* ≤ 0.05, ** *p* ≤ 0.01. (**c**) Transmission electron microscopy was performed on *LRG1* overexpressing (7m8-LRG1) and control (7m8-null) retinas at P10 following intraperitoneal delivery of the tracer HRP. Areas of the leading edge microvessels were analyzed. Images show transverse sections through representative microvessels in lower magnification (**left**) and higher magnification (**right**). Lumen (L), endothelial cell (E), HRP-filled intraendothelial vesicles <100 nm (arrows), empty intraendothelial vesicles <100 nm (arrowheads), HRP-filled intraendothelial vesicles >100 nm (star). Quantification of HRP-filled vesicles <100 nm in diameter (arrows) which were associated with the plasma membrane revealed significantly higher number of vesicles in *LRG1* overexpressing eyes compared to controls. Data from a total of 48 retinal vessels and 6 eyes. Unpaired *t*-test. Mean ± SEM. * *p* ≤ 0.05. Scale bars in overview images 2 μm and 0.1 μm in magnified images.

With the expression and distribution of tight junction proteins apparently undisturbed, we investigated indicators of increased transcellular permeability. Plasmalemma vesicle-associated protein (PLVAP) is a component of caveolae, fenestrae and trans-endothelial channels and is expressed at low levels in developing retinal vessels and is then lost as vessels stabilize and mature [48]. In the adult CNS, the induction of PLVAP is used as a marker of pathological breakdown of the barrier [49]. PLVAP expression was very low in control P5 retinal vessels, both in the central retina and at the leading edge (Figure 5a), in line with the low *Plvap* transcription levels found in the scRNAseq analysis (Figure 1). In eyes that had undergone LRG1 administration, PLVAP expression was significantly higher, suggesting that LRG1 interfered with the developmental downregulation of PLVAP. We next looked at the expression of the lipid transporter major facilitator superfamily domain containing protein 2a (MFSD2A) which correlates positively with the formation of functional CNS vascular barriers in the brain and the retina where it is involved in suppressing endothelial transcellular transport [3,4]. At P5, following LRG1 introduction, MFSD2A expression in the retinal vasculature was significantly reduced at the leading edge compared to control eyes whilst its high expression throughout the central parts of the retinal vasculature remained unchanged (Figure 5b). These data suggested that exogenous LRG1 interfered with the formation of a functional BRB, leading to continued transcellular permeability at the leading edge, which is normally shut down by P10 [3]. To examine this further, we performed transmission electron microscopy on HRP-perfused retinal vessels at P10 and quantified HRP-containing vesicles in the microvascular endothelium of the leading edge. The developing retinal vascular endothelium was rich in cytoplasmic vesicles. We focused our analysis on vesicles with apparent transcytotic activity relevant to this study, namely HRP-positive caveolae (shown to transport albumin across the blood–brain barrier (BBB) and BRB [43]) attached or in close vicinity (<200 nm) of the luminal plasma membrane. In control P10 retinas, the number of HRP-containing caveolae in leading edge endothelial cells was very low (Figure 5c). In contrast, *LRG1* overexpressing retinas exhibited significantly higher numbers of such transcytotic endothelial vesicles than controls, indicating that exogenous LRG1 prevented the developmental establishment of this aspect of the BRB.

### 3.5. LRG1 Gene Delivery Reduces the Expression of Connexin43

**Figure 6:**
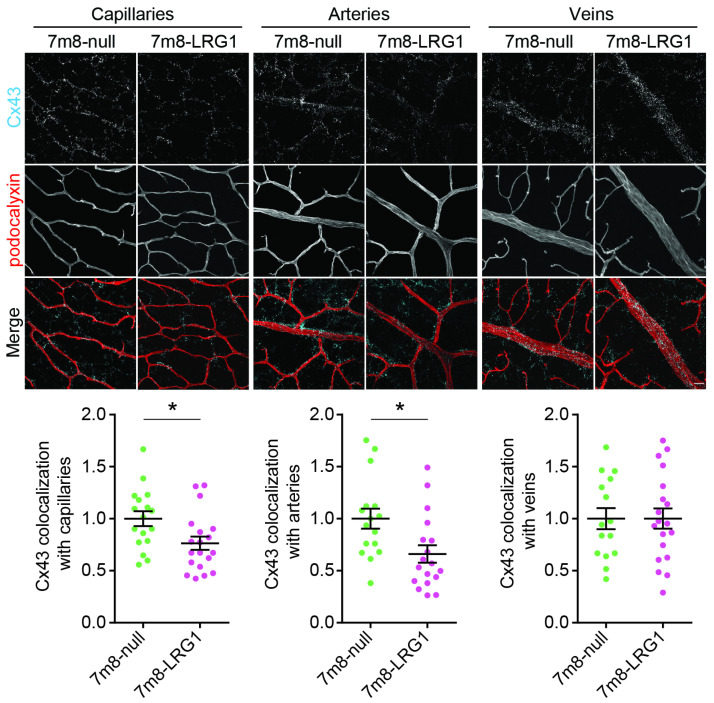
*LRG1* gene overexpression reduces expression of Cx43 on arteries and superficial capillaries. Representative micrographs of retinal vasculature from P16 eyes treated with *LRG1* overexpression (7m8-LRG1) or null vector (7m8-null). The proportion of the vessels overlapping with Cx43 staining was quantified and shows a significant reduction in Cx43 on arteries and the capillaries of the superficial plexus in *LRG1* overexpressing retinas; *n* = 15–19 eyes per group. Unpaired *t*-test. Mean ± SEM of *n* ≥ 3 independent experiments. * *p* ≤ 0.05. Scale bar 25 μm.

Having established that LRG1 modifies pericyte expression and transcellular retinal vascular permeability, we next examined if LRG1 affects the expression of connexin43 (Cx43) as dysfunctional pericytes are associated with defective gap junction formations [50,51]. In the P6 mouse retina, scRNAseq analysis showed expression by endothelial cells, pericytes and astrocytes (see *Gja1*, Figure 1). Following AAV-mediated *LRG1* gene delivery into WT P2 mouse retinas and harvesting at P16 we co-stained the vasculature with podocalyxin and the Cx43 gap junction protein. Image analysis revealed that *LRG1* overexpression in the retina leads to a reduction in the expression of Cx43 in the superficial capillaries and arteries, whereas no change was observed in veins (Figure 6). We did not find Cx43 expression in the deep vessel plexus.

### 3.6. LRG1 Reduces Microvascular Density and Vessel Size of Deep Capillaries and Veins

**Figure 7:**
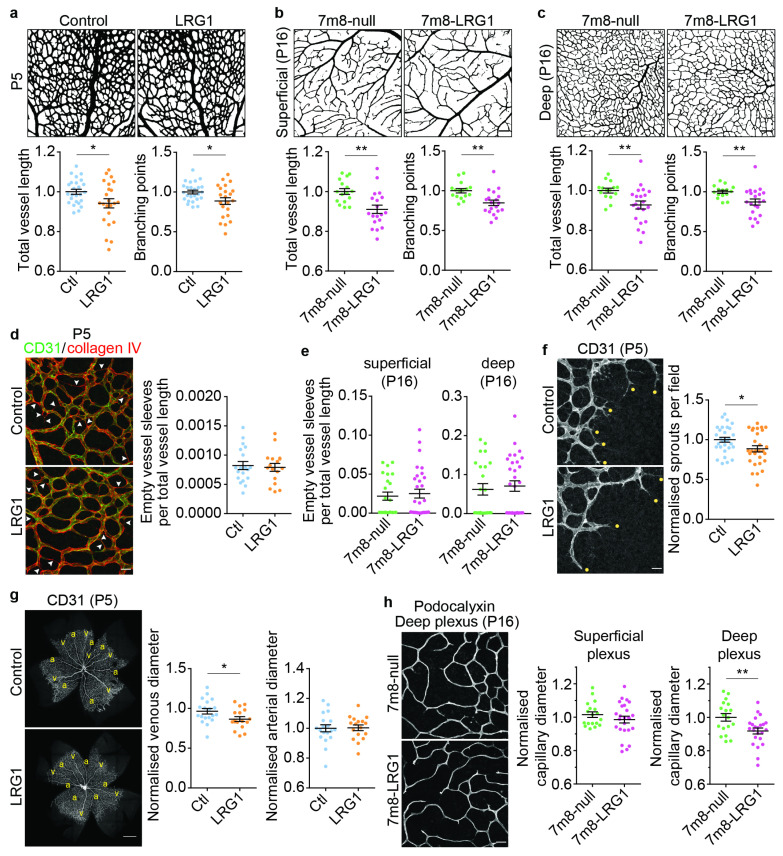
LRG1 alters the vascular growth pattern. Reduced retinal vessel density in LRG1 protein-treated retinas at P5 (**a**) and in *LRG1* overexpressing retinas at P16 (**b**,**c**); *n* = 23–25 eyes per group (**a**), *n* = 16–19 eyes per group (**b**), *n* = 15–21 eyes per group (**c**). Scale bars 100 μm. (**d**) Representative image of the superficial plexus at P5 showing empty (non-endothelialised, collagen IV positive) vessel sleeves (arrow heads). Scale bar 25 μm. Quantification reveals no difference in the amount of vessel regression at P5 between control and LRG1 protein treatment (*n* = 17–19 eyes per group), and at P16 (**e**) in the superficial plexus and deep plexus between control (7m8-null) and *LRG1* overexpressing (7m8-LRG1) retinas; *n* = 23–30 eyes per group. (**f**) Images and dot plot of quantified data showing reduced number of vessel sprouts per field at the leading edge in LRG1-treated retinas at P5; *n* = 29–30 eyes per group. Scale bar 25 μm. (**g**) Representative micrographs and dot plots showing significant reduction in venous, but not arterial, vessel calibre at P5 in LRG1-treated retinas. Arteries (a), veins (v); *n* = 17–19 eyes per group. Scale bar 500 μm. (**h**) Representative images of the capillaries of the deep vessel plexus (scale bar 25 μm). Dot plot of significant reduction in capillary calibre in the deep vessel plexus at P16 in *LRG1* overexpressing retinas, but no change in capillary thickness of the superficial plexus; *n* = 18–25 eyes per group. Unpaired *t*-test or Mann–Whitney test depending on normality. Mean ± SEM, * *p* ≤ 0.05, ** *p* ≤ 0.01.

Next, we examined if the observed effects of LRG1 resulted in any anomalies in the vascular growth pattern of the superficial and deep plexuses. The presence of LRG1 reduced total tubule length and the number of branching points (Figure 7a–c). This also resulted in a reduced density of the vascular network at P5 after injection of LRG1 protein, as well as on P16 in *LRG1* overexpressing retinas. A change in vascular density may be caused by a decrease in vessel sprouting or an increase in vessel pruning, whereby the regression of selected vessel branches occurs to remodel the vasculature into a hierarchical and functional vascular pattern from around P5 [52]. To assess pruning, we quantified empty basement membrane sleeves [52] and this revealed no significant difference between LRG1-treated retinas and controls or AAV-mediated *LRG1* gene delivery and controls (Figure 7d,e). However, fewer vessel sprouts were detected at the leading edge on P5 in the LRG1-treated group, pointing to a reduction in sprouting angiogenesis as a potential cause for the lower vascular density (Figure 7f). Furthermore, LRG1 also significantly reduced the diameter of the retinal veins and capillaries of the deep retinal plexus, but not of arteries or superficial capillaries (Figure 7g,h).

## 4. Discussion

During physiological angiogenesis, newly formed vessels undergo a series of changes to become fully stable and functional, including the recruitment of pericytes. Pericytic investment represents a crucial step in vessel stabilization and maturation and, in the retina, is necessary for the formation and maintenance of a fully functioning inner blood–retinal barrier [53]. LRG1 has been shown to promote the formation of dysfunctional vessels in various disease conditions [8,12] and to compromise pericyte coverage of tumour vessels [24]. Here, we hypothesized that in the absence of any other pathological drivers its introduction during developmental angiogenesis would impact on vascular structure and function. We demonstrated that the introduction of exogenous LRG1 reduced pericyte coverage and pericyte expression of NG2 in the developing retinal vasculature. Consistent with this, we observed in the OIR model of pathogenic retinal neovascularization, where *Lrg1* is highly upregulated [11], that deletion of *Lrg1* resulted in increased NG2 expression. These data show that, in addition to cancer vessels [24], LRG1 also impacts on pericytes in a non-oncological setting.

Pericyte recruitment to the developing vessels is essential for the assembly of the vascular basement membrane because the expression and deposition of extracellular matrix by endothelial cells and pericytes is only induced if both cell types are in close contact [5]. Our observation of reduced basement membrane vessel sheathing after introduction of exogenous LRG1 is in line with our previous finding of increased collagen IV coverage in tumours in *Lrg1*^−/−^ mice [24], and could be due to LRG1 decreasing pericyte coverage and disrupting the close apposition of endothelial cells and pericytes.

In addition to its role in the assembly and maintenance of the basement membrane, extensive pericyte coverage inhibits vesicular activity of CNS endothelial cells and consequently limits BBB transcellular permeability [3]. Under normal conditions the leakage observed from sprouting vessels during retinal vascular development is not believed to be due to a lack of tight junctions, as they form early, but to high levels of transcytosis. Indeed, the functional formation of the BRB correlates with downregulation of caveolar transcytosis [4]. Two key proteins involved in endothelial vesicle trafficking and the regulation of transcytosis are PLVAP and MFSD2A. PLVAP is downregulated during vascular maturation and is virtually absent in retinal vessels from day P10, coinciding with the formation of a functional BRB [54]. Significantly, gene expression profiling that originally identified *Lrg1* as the most upregulated gene in pathogenic retinal vessels also revealed that the second most induced gene was *Plvap* [11] functionally linking these two genes. In contrast, MFSD2A is an important negative regulator of transcytosis at the BRB and BBB and its expression in retinal endothelia is essential to suppress transcytosis at the developing BRB [3,4]. These findings are consistent with the observed increase in vesicular activity and point to LRG1 having its greatest effect on transendothelial permeability, possibly through its impact on pericyte–endothelial cell cross talk.

In addition to a reduction in pericyte coverage, we found that LRG1 decreases the expression of pericytic NG2 which might have impacted several of our findings. Indeed, germline NG2-KO, macrophage-specific NG2-KO, or pericyte-specific NG2-KO lead to reduced pericyte coverage of endothelial cells in tumours [55,56,57] and, as in our study, to a reduction in basement membrane expression. Furthermore, similar to our observations, loss of NG2 in other settings has also been reported to impair vascular growth and density including in tumours [55,58,59], transplanted tissue [60] and models of ocular angiogenesis [61] and to affect vessel calibre [55]. Our observation that LRG1 affects endothelial junctional molecule expression is also consistent with a reduction in NG2 expression, as NG2 has been shown to enhance junction formation [56], and promote the expression of CD31 in tumour-associated vessels [55].

The role played by NG2 in shaping pericyte function, and their relationship with endothelial cells, is not fully defined but integrin signalling has been proposed as a possible underlying mechanism. Thus, NG2 expressed on the cell surface of pericytes can activate β1 integrin signalling in endothelial cells which is involved in pericyte recruitment to the vessel, vessel growth and endothelial cell junction formation [57,62]. In addition, NG2 contains binding sites for several proangiogenic growth factors [63], and is involved in the formation of the basement membrane by serving as a cell surface ligand for collagen VI [64] which in turn binds collagen IV [65]. However, whether a relative reduction in NG2 expression, as opposed to a complete ablation, is sufficient to cause the effects we observe remains to be determined.

LRG1 also decreased the expression of the gap junction protein Cx43 in arteries and capillaries but not veins, which may result in compromised endothelial cell–pericyte crosstalk and contribute to vessel dysfunction. In support of this, Cx43 has been shown to be involved in regulating retinal vessel diameter and blood flow [51,66] and loss of expression correlates with a defective retinal vasculature in diabetes [67,68]. Furthermore, Cx43 is necessary for the activation of latent TGFβ and endothelium-induced mural cell differentiation [50], pointing to its key role in vascular homeostasis.

We also observed that LRG1 reduced vascular density and calibre, both of which may be influenced by TGFβ signalling [69,70,71,72,73]. In accordance with the observed changes, we previously reported that tumour vessel diameter in *Lrg1*^−/−^ mice is larger than in wildtype mice [24]. This could be a result of LRG1 modulating TGFβ signalling in endothelial cells by binding to TGFβ accessory receptor endoglin and switching TGFβ signalling towards the Alk1—Smad1/5/8 pathway [11]. Our outcomes are consistent with previous studies showing that a shift in TGFβ-signalling towards the ALK1-smad1/5/8 pathway can impact on vessel branching and calibre [74,75,76]. Interestingly, LRG1 has been shown to induce VEGF expression [77], but the changes in vessel patterning we observe are not consistent with VEGF-mediated effects like microaneurysms, tortuosity, vessel dilation and the formation of neovascular vessels which have been described in VEGF overexpressing transgenic mice [78,79].

The structural and functional vessel alterations in our study may be exacerbated by changes to the junction-associated endothelial cell proteins CD31 and VE-cadherin, as these are known to be required for vascular tubulation and the formation and integrity of cell junctions [47,80]. VE-cadherin affects important angiogenic functions such as vessel protrusion at the angiogenic fronts and dynamic alignment of endothelial cells to alterations in shear stress including actin cytoskeleton rearrangements [81]. As such it is interesting that loss of VE-cadherin was only seen at the angiogenic front and in the deep plexus, but not in the more mature vasculature in the retinal centre at P5 or in the superficial plexus at P16. In accordance with these results, an increase in VE-cadherin expression and improved vascular function in B16F0 tumours following treatment with a LRG1-blocking antibody has been reported [24]. However, despite changes in VE-cadherin we did not observe any changes in the tight junction proteins claudin-5 and occludin. Notably, fully developed tight junctions are found very early during retinal vascularization in budding vessels from postnatal day 1 [4]. VE-cadherin regulates homotypic endothelial cell adhesion and thereby also paracellular flux in many vascular beds [82], and whilst VE-cadherin is critical for the assembly of the junctional complex in developing vessels [83], its loss in the CNS, unlike other organs, is not associated with increased paracellular permeability [84]. This points to a different and possibly more robust regulation of barrier properties in the CNS, including the retina, and might explain why the partial reduction in VE-cadherin expression which we observed did not affect the tight junctions that had developed sufficiently to create a functional paracellular barrier.

The LRG1-mediated changes to the retinal vasculature described above were not uniform throughout the retina and most likely reflect the period and duration of LRG1 exposure. For example, most of the changes observed after injecting the AAV7m8-LRG1 overexpression vector were stronger in the deep vessel plexus than in the superficial plexus. This differential effect may be the consequence of the deep plexus being exposed to higher LRG1 concentrations due to its position in between the *LRG1*-transduced outer and inner retinal nuclear layers. More likely, however, is that, by the time *LRG1* translation is maximal, the superficial plexus will be well established whilst the deep vessel plexus will still be developing. On the other hand, some observed changes at P16, such as the alteration of CD31 expression, were more pronounced in the superficial plexus, which indicates that LRG1 not only affects the vasculature while it is actively growing, but also during the subsequent remodelling phase.

To conclude, this study shows that the introduction of LRG1 during retinal blood vessel development is sufficient to drive the formation of structurally and functionally abnormal vessels, and that the pericyte–endothelial cell axis is a key target of LRG1 vasculopathic activity. The presence of LRG1 in many pathologies suggests, therefore, that it is a major factor responsible for failed physiological angiogenesis in disease. These data pave the way for further studies to investigate the wider implications of LRG1-mediated vascular dysfunction and add to the increasing evidence that LRG1 is a therapeutic target of considerable potential.

## Data Availability

Data will be available from the corresponding author upon reasonable request.

## Supplementary Materials

The following supporting information can be downloaded at: https://www.mdpi.com/article/10.3390/cells14080593/s1, Figure S1: Verification of vector gene expression; Figure S2: Deletion of *Lrg1* increases the expression of NG2 in oxygen-induced retinopathy (OIR); Figure S3: LRG1 does not significantly alter the expression of PDGFRβ in pericytes; Figure S4: LRG1 reduces the expression of junction associated protein CD31; Figure S5: Retinal mouse *Tgfβ1* mRNA expression; Supplementary Methods [15].
