# Supplementary material for: LRG1 Alters Pericyte Phenotype and Compromises Vascular Maturation
Source: Cells. 2025 Apr 14;14(8):593. doi: 10.3390/cells14080593 (PMC12026257; doi:10.3390/cells14080593)
Supplement: Supplementary file 1 [file cells-14-00593-s001.zip › cells-3495049-supplementary.pdf]

# Supplementary information

## LRG1 alters pericyte phenotype and compromises vascular maturation

Alexandra E Hoeh[1,*], Jui-Hsien Chang[1], Ronja S Mueller[1], Mark Basche[1,2], Alessandro Fantin[3], Anastasios Sepetis[1], Giulia De Rossi[1], Athina Dritsoula[1], Robin Ali[1,2], Patric Turowski[1], Stephen E Moss[1,4], John Greenwood[1,4]

[1] Institute of Ophthalmology, University College London, London, UK

[2] Ocular Cell and Gene Therapy Group, Centre for Gene Therapy and Regenerative Medicine, King's College London, London, UK

[3] Department of Biosciences, University of Milan, Milan, Italy

[4] These authors contributed equally

* Corresponding author:
Alexandra Eva Hoeh
Institute of Ophthalmology,
University College London,
London
a.hoeh@ucl.ac.uk

This file includes:

Supplementary figures S1 – S5

Supplementary methods

Supplementary references

## Supplementary Figure S1

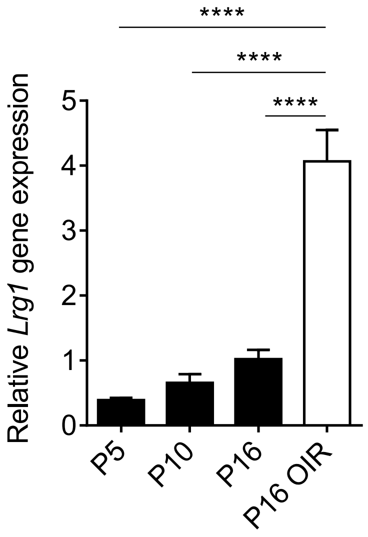


**Supplementary Fig. S1 Retinal mouse *Lrg1* expression during vascular development**
Quantification of mouse *Lrg1* mRNA in retinal lysates of wild type pups on P5, P10 and P16 at room air, and at P16 in oxygen induced retinopathy (OIR). Gene expression was normalised to *Gapdh*. *Lrg1* gene expression is upregulated in retinas of P16 OIR pups compared to room air controls (Ctl). Mean + SEM of n=3 eyes/group. ANOVA and Dunnett's multiple comparisons test. **** $P \leq 0.0001$.

## Supplementary Figure S2

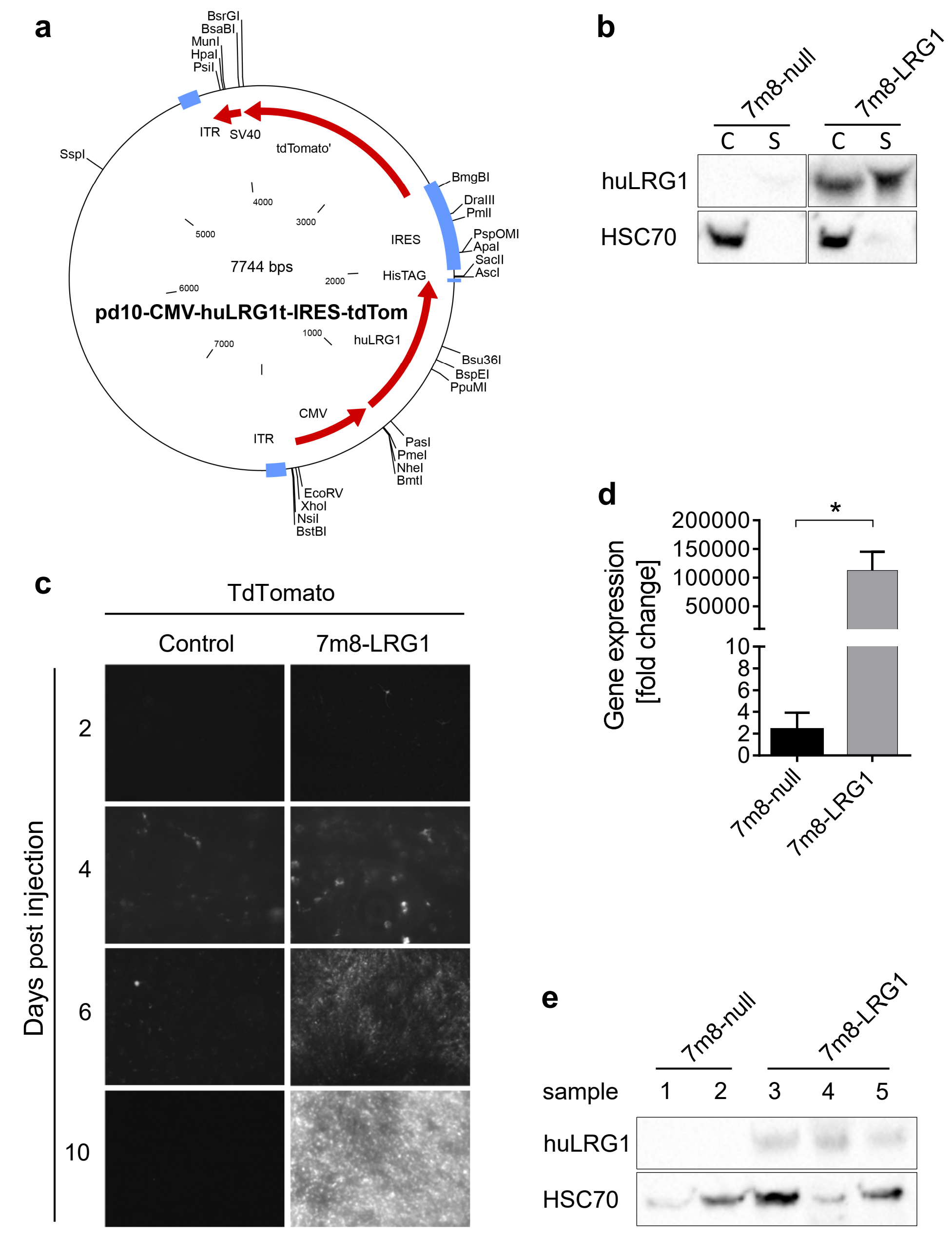


**Supplementary Fig. S2 Verification of vector gene expression**
**a** The human *LRG1* overexpression vector construct is based on a pd10 backbone plasmid which contains the AAV2 (adeno-associated-vector 2) ITRs (inverted terminal repeats) and was packaged within a 7m8 capsid. A CMV (cytomegalovirus) promoter was used to drive the expression of the *LRG1* transgene. It was constructed as a bicistronic vector with an internal ribosomal entry site (IRES) which allows simultaneous expression of the human his-tagged LRG1 and the reporter tdTomato from the same mRNA transcript. **b** Western blot of cell lysates (C) and supernatant (S) of transduced HEK293T cells demonstrates LRG1 expression and secretion. **c** TdTomato expression in retinal flatmounts after intravitreal injection of the *LRG1* overexpression vector (7m8-LRG1) is first seen 4 days after injection and increases to high levels between day 6 and 10. Image exposure time had to be reduced from 500ms to 150ms on day 10. **d** Quantification of *LRG1* mRNA in transduced retinas. Retinas were harvested 14 days after intravitreal injection of 7m8-LRG1 or control (7m8-null) on P16. High retinal *huLRG1* gene expression after 7m8-LRG1 injection. Gene expression was normalised to *Gapdh* and the null vector control. Unpaired t-test. Mean + SEM. **e** Western blot for LRG1 in whole eye lysates of five different eyes shows *huLRG1* expression in eyes which were transduced with the *LRG1* overexpression vector, but not in the control eyes.

## Supplementary Figure S3

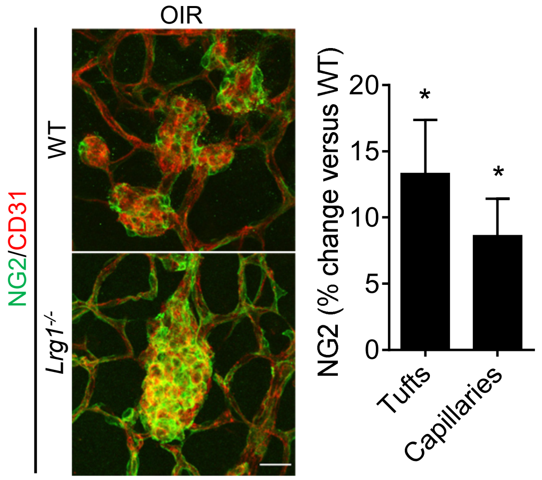


**Supplementary Fig. S3 Deletion of *Lrg1* increases the expression of NG2 in oxygen-induced retinopathy (OIR)**
OIR was induced in the offspring of heterozygous *Lrg1*$^{-/+}$ breeders. Comparison of NG2 pixel intensity in P16 retinas from wild type (WT) and *Lrg1*$^{-/-}$ pups revealed higher expression of NG2 on neovascular tufts and the capillaries of the superficial plexus in *Lrg1*$^{-/-}$ pups. Unpaired t-test. Mean + SEM of n≥3 independent experiments. * $P \leq 0.05$. Scale bar 25 µm.

## Supplementary Figure S4

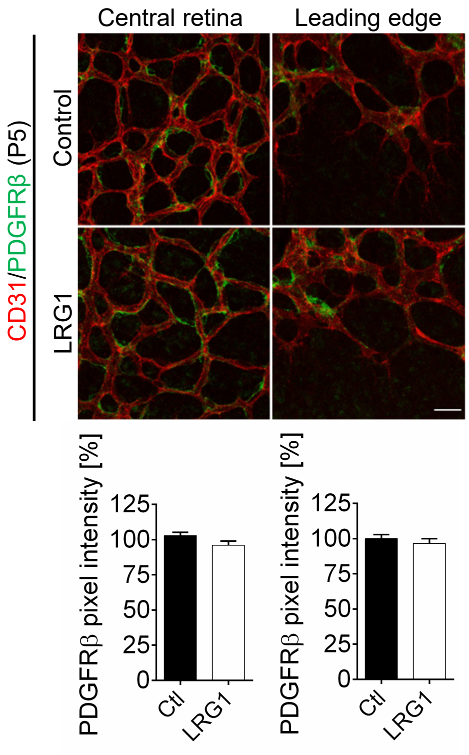


**Supplementary Fig. S4 LRG1 does not significantly alter the expression of PDGFRβ in pericytes**

In the central retina, LRG1 protein treated retinas showed a small, but insignificant (p=0.07) decrease of PDGFRβ expression in pericytes compared to controls (Ctl). No change of PDGFRβ expression at the leading edge. Unpaired t-test. Mean + SEM of n≥3 independent experiments. Scale bar 25 µm.

## Supplementary Figure S5

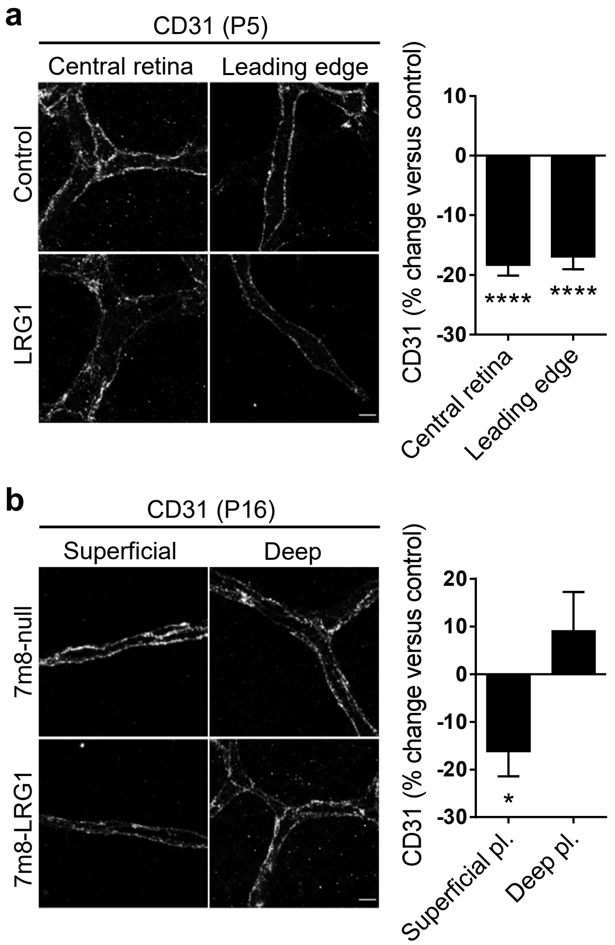


**Supplementary Fig. S5 LRG1 reduces the expression of junction associated protein CD31** Pixel intensity of CD31 was analysed at the endothelial junctions which were delineated with claudin-5 or occludin co-staining for analysis. **a** Significant reduction of CD31 expression at P5 after LRG1 protein treatment compared with control in the central retina and leading edge. **b** Significant reduction of CD31 expression in the superficial vascular plexus in *LRG1* overexpressing retinas, but no difference was observed in the deep vessel plexus. Unpaired t-test. Mean + SEM of n≥3 independent experiments. * $P \leq 0.05$, **** $P \leq 0.0001$. Scale bars 5 μm.

## Supplementary methods

**Quantitative PCR (qPCR)** RNA was extracted using the RNeasy Mini Kit (74104, Qiagen) and reverse transcribed (Quantitect reverse transcription kit, 205313, Qiagen). The quantitative polymerase chain reaction was performed with Power SYBR Green PCR master mix (4309155, Applied Biosystems). Gene expression was normalised to the housekeeping gene glyceraldehyde-3-phosphate dehydrogenase (*Gapdh*) and the control using the double delta Ct method. The following primers were used: *huLRG1* forward primer CAGCGACCAAAAAGCCCAG, reverse primer ATTTCGGCAGGTGGTTGACA; *mLrg1* forward CCATGTCAGTGTGCAGATTC, reverse AAGAGTGAGAGGTGGAAGAG; *mGapdh* forward ACTGAGGACCAGGTTGTCTCC, reverse CTGTAGCCGTATTCATTGTCATACC.

**Western blot** The tissue was homogenized and lysed in NP-40 buffer with phosphatase and protease inhibitors, and proteins were separated in a polyacrylamide gel and transferred onto a PVDF membrane. After blocking in 5% milk, the membranes were incubated overnight with the following primary antibodies: magacizumab (humanized IgG4 antibody against LRG1)[2] and mouse monoclonal Anti-Hsc70 antibody (SAB3701436, Sigma-Aldrich), followed by horseradish peroxidase (HRP)-conjugated secondary antibodies. Heat shock cognate 71 kDa protein (Hsc70) was used as loading control. The chemiluminescent signal was acquired with the ChemiDoc MP imaging system (Bio-Rad) using the ImageLab 5.2.1 software. ImageJ software was used for densitometry.

## Supplementary references


1. Kallenberg, D. et al. A Humanized Antibody against LRG1 that Inhibits Angiogenesis and Reduces Retinal Vascular Leakage. doi:bioRxiv 10.1101/2020.07.25.218149 (2021).
